# Low referral completion of rapid diagnostic test-negative patients in community-based treatment of malaria in Sierra Leone

**DOI:** 10.1186/1475-2875-10-94

**Published:** 2011-04-17

**Authors:** Anna Thomson, Mohammed Khogali, Martin de Smet, Tony Reid, Ahmed Mukhtar, Stefan Peterson, Johan von Schreeb

**Affiliations:** 1 Division of Global Health, Karolinska Institutet, Stockholm, Sweden; 2 Médecins Sans Frontières Operational Centre Brussels, Belgium; 3 Médecins Sans Frontières Operational Centre Brussels, Bo, Sierra Leone

## Abstract

**Background:**

Malaria is hyper-endemic and a major public health problem in Sierra Leone. To provide malaria treatment closer to the community, Médecins Sans Frontières (MSF) launched a community-based project where Community Malaria Volunteers (CMVs) tested and treated febrile children and pregnant women for malaria using rapid diagnostic tests (RDTs). RDT-negative patients and severely ill patients were referred to health facilities. This study sought to determine the referral rate and compliance of patients referred by the CMVs.

**Methods:**

In MSF's operational area in Bo and Pujehun districts, Sierra Leone, a retrospective analysis of referral records was carried out for a period of three months. All referral records from CMVs and referral health structures were reviewed, compared and matched for personal data. The eligible study population included febrile children between three and 59 months and pregnant women in their second or third trimester with fever who were noted as having received a referral advice in the CMV recording form.

**Results:**

The study results showed a total referral rate of almost 15%. During the study period 36 out of 2,459 (1.5%) referred patients completed their referral. There was a significant difference in referral compliance between patients with fever but a negative RDT and patients with signs of severe malaria. Less than 1% (21/2,442) of the RDT-negative patients with fever completed their referral compared to 88.2% (15/17) of the patients with severe malaria (RR = 0.010 95% CI 0.006 - 0.015).

**Conclusions:**

In this community-based malaria programme, RDT-negative patients with fever were referred to a health structure for further diagnosis and care with a disappointingly low rate of referral completion. This raises concerns whether use of CMVs, with referral as backup in RDT-negative cases, provides adequate care for febrile children and pregnant women. To improve the referral completion in MSF's community-based malaria programme in Sierra Leone, and in similar community-based programmes, a suitable strategy needs to be defined.

## Background

Malaria is one of the leading causes of mortality and morbidity among children in Sub-Saharan Africa. The disease causes nearly 800,000 deaths per year, most of which are in children under five years of age [1]. Sierra Leone has one of the highest under-five mortality rates in the world, corresponding to 269 deaths per 1,000 live births [2]. Malaria is hyper-endemic and one of the main killers in children under five years of age in the country [3]. Due to the high chloroquine resistance, the Ministry of Health and Sanitation (MOHS) in Sierra Leone in 2004 changed the first-line therapy of the national malaria protocol to artemisinin-based combination therapy (ACT) [4]. Based on the new WHO recommendations, the MOHS has recently decided to increase the use of rapid diagnostic tests (RDTs), particularly in settings where capacity for microscopic diagnosis is limited [5]. Access to health care is still a major problem for the population in Sierra Leone. Several factors such as poverty, lack of transport, distance and user fees contribute to delayed care-seeking for febrile children, especially in rural areas [6].

Médecins Sans Frontières (MSF) has been working in Sierra Leone since 1995. In collaboration with the MOHS in Bo and Pujehun districts, MSF provides free primary and secondary health care. In 2008 malaria accounted for over 40% of all consultations in the MSF-supported clinics and health posts in Bo and Pujehun districts[7]. At the end of 2007, MSF introduced Community Malaria Volunteers (CMVs) in order to provide free and effective malaria treatment closer to the community. The CMVs were identified and selected by the community based on three main criteria: they should be able to read and write; they should speak English and they should be respected and liked in the community. Early 2008 the selected CMVs were given three days of training on diagnosis and treatment of malaria using RDTs (Paracheck^®^), ACT (artesunate-amodiaquine) and paracetamol. They were also trained to recognize and ask for signs of severe malaria (e.g. convulsions, unconsciousness, repeated vomiting, little or no output of urine, diarrhoea, dehydration and anaemia). The training was both theoretical and practical, including a session on how to perform and read RDTs.

By June 2009, a total of 136 CMVs operated in 130 villages within MSF's catchment area treating children between three months and five years of age and pregnant women, except for those in the first trimester of their pregnancy, for malaria. Two CMV supervisors with motorcycles oversaw the CMV activities and carried out random supervision visits and monthly meetings where drugs and RDTs were distributed, statistics were collected and general issues related to the CMVs' work were discussed. The CMVs were meant to refer all cases with signs of severe malaria and those with complaints of fever but with a negative RDT to the nearest health facility, located within a range of three to 18 kilometres from the villages where the CMVs were based.

A key component of community-based malaria care, using minimally-trained volunteers, is an effective referral system, to ensure that patients with severe malaria or other illnesses receive treatment. This has been discussed in several studies, describing various referral patterns and with referral completion rates varying from 37.5% to 87% [8-10]. Before this study there had been no follow up of CMV referrals in MSF's community-based malaria programme, and there was a need to evaluate the programme defining total referrals, referral completion and outcome of referrals. The findings would be of relevance for all MSF projects focusing on malaria, as well as for other organizations and programmes involved in community-based treatment of malaria. Thus, the aim of this study was to determine the referral rate, compliance and diagnostic profile of referrals from CMVs to the MSF supported health structures in two districts in Sierra Leone.

## Methods

### Setting

This study took place in MSF's operational area in rural areas of Bo and Pujehun districts, Sierra Leone, with an estimated target population of 302,000. These districts were hyper-endemic malaria zones with high transmission rates all year, especially during the rainy season from June to October. The CMV catchment area consisted of 130 villages. There were one or two CMVs in each village depending on population size. The CMVs were divided into 16 networks, each linked to a health centre or a health post for supervision and referral. These health structures were under the responsibility of the MOHS but jointly managed by MSF. Health care was provided for free, and MSF covered costs for staff salaries, drugs and medical materiel and provided supervision. Health posts were provided with partial support (limited number of drugs for children less than 15 years of age and pregnant women). The distance from the villages to the nearest MSF supported health structure varied from three to over 18 kilometres. Apart from the MSF-supported health structures there were no other major care providers (e.g. private clinics or drug vendors).

### Population

**Figure 1:**
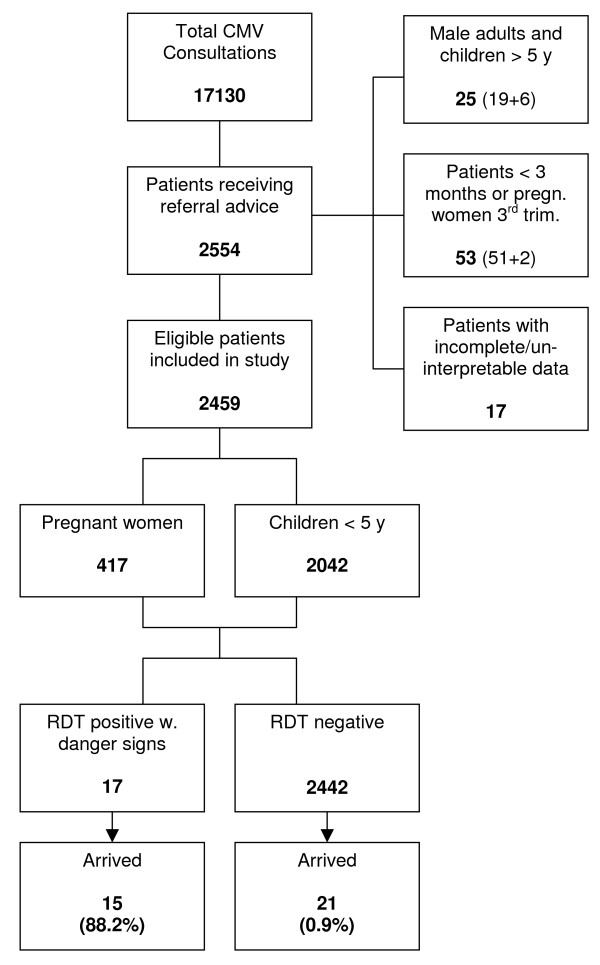
**Flowchart showing study inclusion and referral outcome**.

The study population included all children between three and 59 months of age and pregnant women in their second or third trimester who were recorded in the CMV recording forms as referrals. The CMVs had been trained to refer all patients with signs of severe malaria and a positive RDT and all patients with complaints of fever but with a negative RDT. Children less than three months of age and pregnant women in their first trimester, who would always be referred due to contra-indications of ACT, were excluded. Patients with incomplete or un-interpretable data and repeated referrals were also excluded from the study (Figure 1).

### Study procedures and data collection

Data on patients seen by CMVs were entered in a recording form and included basic demographic information, RDT result, treatment given and date of and indications for referral. The CMVs were instructed about the importance of accurate data recording. In addition, a referral follow-up register was introduced in June 2009 into all 16 MSF-supported health structures linked to a CMV network. The health centre/health post personnel were trained on how to fill it in properly and were instructed to ask each patient arriving at the structure whether or not he/she was referred from a CMV. The health structure register contained basic demographic information as well as final diagnosis and treatment. The CMV recording forms were collected during monthly supervision visits by two CMV-supervisors. All records from CMVs and referral follow-up registers were matched and demographic data, date of arrival, name of health structure and name of the referring CMV were compared. If all the above data matched, the referral was confirmed as being completed. For each completed referral, final diagnosis and time of arrival at the health structure (> or < 24 hrs) were recorded. The data collection was overseen by the field investigator, who was able to give regular support to CMV supervisors, health centre/health post nurses and others involved in this study at field level. All data were routinely collected from the CMVs and the health structures. Raw data were entered, validated and encoded in Sierra Leone by the field investigator and mission epidemiologist, with the help of three data-encoders.

### Analysis

The analysis of record reviews was carried out over a three months period, from the 1^st ^of September to the 30^th ^of November 2009. Every village and CMV had an assigned code that was used when entering and crosschecking the data. All patients included in the study were assigned an identification number to facilitate study-related documentation and ensure confidentiality. The principal investigator, who had previously worked as field coordinator in MSF's project, did the analysis at Karolinska Institutet in Stockholm. All data were coded, entered into the data analysis software and double-checked to ensure consistency and accuracy. Data was analysed for frequencies of referral completion and stratified by sex, age and RDT-status using EpiInfo, version 3.5.1.

### Ethics

The routinely collected programme data that was used in this study was handled with confidentiality. The study was approved by MSF's Ethics Review Board. Permission to carry out the study had previously been granted from the MOHS in Sierra Leone.

## Results

### Demographic characteristics

During the study period the CMVs saw a total of 17,130 patients with complaints of fever. Out of these patients 12,204 (71%) were eligible children and 4,901 (28%) were pregnant women. Out of 2,554 patients registered as referred, 25 patients (1%) were male adults (19) or children above five years of age (6), not fitting the inclusion criteria. Seventeen patient-entries were excluded from the survey due to incomplete or un-interpretable data and 53 referrals were excluded as they were less than three months of age (51) or pregnant women in their first trimester (2). Out of the 2,459 remaining referrals, 2,042 (83%) were children under five years of age and 417 (17%) were pregnant women (Table 1). Out of the 2,042 children 1,102 (54%) were male and 940 (46%) were female. The mean age for the children was 2.4 years. The age of the pregnant women varied from13 to 49 years with a mean of 25 years (Table 2).

**Table 1:** Total CMV consultations over the defined study period

CMV consultations	*n*	RDT+	(%)	RDT-	(%)
< 5 years	12204				
Pregnant	4901				
Others	25				

Total	17130	14235	(83.0)	2895	(17.0)

**Table 2:** General characteristics of eligible study population

Children < 5 y		M	F	Tot.	(%)
Total		1102	940	2042	(83.0)
	< 1 y	131	128	259	(10.5)
	1 y	184	170	354	(14.4)
	2 y	274	233	507	(20.6)
	3 y	234	195	429	(17.4)
	4 y	201	136	337	(13.7)
	5 y	78	78	156	(6.3)

**Pregnant women**				***n***	**(%)**

Total				417	(17.0)
	> 15 y			3	(0.1)
	15-19 y			66	(2.7)
	20-24 y			137	(5.6)
	25-29 y			121	(4.9)
	30-34 y			58	(2.4)
	35-39 y			21	(0.9)
	40-44 y			7	(0.3)
	45-49 y			4	(0.2)

**Total referred**				***n***	**(%)**

				2459	(100.0)

### Overall referral rate and reasons for referral

Out of the 17,130 patients seen by a CMV, 2,554 (14.9%) were referred to the nearest MSF-supported health structure. A total of 17% (2,093) of the children under five and 8.5% (419) of the pregnant women were given a referral advice. The referral rate varied from 15.7% (876/5,566) to 13.4% (796/5,937) over the three study months. Out of the total referrals, 99.3% (2,442/2,459) had a negative RDT while 17 (0.7%) tested positive, and were referred with signs of severe malaria. The main referral reason for RDT-negative patients was indicated as "fever" (99.7%, 2,436/2,442). RDT-positive patients were referred for different reasons, such as "complicated", "fever/vomiting", "fever/cough" and "high fever".

### Referral completion rate and determinants of referral compliance

**Figure 2:**
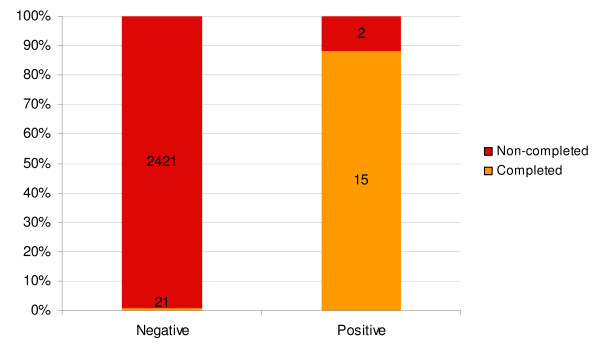
**Referral completion by RDT-status**.

During the full study period only 36 out of 2,459 patients completed their referral, indicating a referral completion rate of 1.5%. The referral completion rate varied from 2.3% (19/812) to 0.5% (4/779) by study month. Referral completion rate ranged from 0% (0/21) to 18.8% (12/52) across the 16 different networks. The completion rate for children was 1.6% (33/2,042) whereas only three (0.7%) of the 417 referred pregnant women arrived at a health structure. A total of 51.5% (17/33) of completed referrals among children were female and 48.5% (16/33) were male (RR = 1.247 95% CI 0.634 - 2.454). The completion rate varied from 2.7% (7/252) among children less than one year to 0.8% (4/503) for children between two to three years (RR = 3.490 95% CI 1.030 - 11.820). A total of 88.2% (15/17) of patients who tested positive for malaria and were referred with signs of severe malaria completed their referral, while only 0.9% (21/2,442) of patients with a negative RDT arrived at a health structure (RR = 0.010 95% CI 0.006 - 0.015) (Figure 2).

### Outcome of completed referrals and significance of time

The outcome analysis was limited to the final diagnosis, listed in the CMV referral follow-up register books. All patients referred with a positive RDT should have had signs of severe/complicated malaria. According to the CMV referral follow-up registers, a large part (40%, 6/15) of these patients were diagnosed as "malaria". Others were diagnosed as "malaria/anaemia" (20%, 3/15), "malaria/acute respiratory infection (ARI)" (13%, 2/15), "malaria/convulsion" (13%, 2/15), "malaria/vomiting" (6.7%, 1/15) and "malaria/skin infection" (6.7%, 1/15). Out of the patients referred with a negative RDT, the most common diagnosis was "ARI" (38%, 8/21), followed by "common cold" (19%, 4/21), "abscess", "skin infection", "fever of unknown origin", "headache" (9.5%, 2/21 respectively) and "nephritis" (4.8%, 1/21). Out of the 36 patients who completed their referral and arrived at a health structure, 92% (33/36) arrived on the same day or within 24 hours. Three patients (8%) arrived at the health structure after 24 hours (Table 3).

**Table 3:** Final diagnosis of completed referrals

		*n*	(%)		< 24 hours		> 24 hours
RDT negative							
	ARI	8	(22.2)		5		3
	Common cold	4	(11.1)		4		0
	Abscess	2	(5.6)		2		0
	Fever of unknown origin	2	(5.6)		2		0
	Headache	2	(5.6)		2		0
	Skin infection	2	(5.6)		2		0
	Nephritis	1	(2.8)		1		0
RDT positive							
	Malaria	6	(16.7)		6		0
	Malaria/anaemia	3	(8.3)		3		0
	Malaria/ARI	2	(5.6)		2		0
	Malaria/convulsion	2	(5.6)		2		0
	Malaria/vomiting	1	(2.8)		1		0
	Malaria/skin infection	1	(2.8)		1		0

Total		36	(100.0)		33		3

## Discussion

This study showed that very few patients who were given a referral advice from a CMV completed their referral. This is an important operational finding, questioning the quality of care of community-based malaria programmes relying on referral of cases with RDT-negative fever.

The RDT result was found to be a significant determinant of referral completion. Less than one percent of patients with a negative RDT completed their referral, while nearly 90 percent of patients who tested positive for malaria, but could not be treated by the CMV, arrived at a health structure. This finding suggests that patients with signs of severe malaria usually complete their referral.

A large variation of referral completion across the 16 networks was notable. It could possibly be linked to variations in distance, CMV performance or differences in registration and follow-up by the health personnel in the various health structures. Due to the small number of completed referrals, no conclusions could be drawn regarding the outcome or timeliness of referrals.

Limited access to healthcare and lack of trained health workers has increased attention to task-shifting. Community Health Workers (CHWs), trained to recognize and treat simple malaria at village level, are becoming an integral part of many health systems. Research has shown that community-based malaria control programmes can reduce paediatric hospitalisations [11], improve care-seeking behaviour [12] and reduce the workload in health facilities [13]. Still, only a few studies give evidence for reduced mortality [14,15] and additional research is definitely needed in order to guide policy decision-making and implementation [16].

An important factor in the use of CHWs to reduce mortality is completion of their referrals. Referral compliance varies in different contexts and at various levels of health care. In Uganda, 87% of patients referred to health centres by CHWs complied with the referral [10], while compliance among referrals from health centres to hospital was only 28% [17]. Several factors can influence adherence to referrals including access (e.g. transportation, overnight stay) and family dynamics (mother not being the decision maker) [18]. Signs of severity, such as altered consciousness and convulsions, can increase adherence up to three times, while costs (e.g. for lab services) led to four to five times lower adherence [8].

It is difficult to understand why the referral completion rate in MSF's CMV programme was so low. There could be several reasons, as described above, but adequate supervision and training could be one way of improving the referral compliance. Several studies have shown that training is essential in order to ensure a successful community-based malaria programme. For example, training of caretakers, as well as of private drug vendors in Nigeria has lead to improved recognition, treatment and referrals of malaria cases [19,20]. A training programme for CHWs in Mali increased adherence to referrals to an encouraging 87% [9]. Health worker actions, such as giving a referral slip and advising caretakers to go to the hospital immediately, can also improve referral completion[18]. It is, however, important to bear in mind that CHWs require not only training, but also adequate supervision and remuneration [21]. In MSF's programme supervision is a challenge, with only two CMV supervisors overseeing and coordinating the CMV activities.

One possible strategy to reduce the poor referral compliance rate is to narrow the diagnostic criteria for referral so that patients could avoid unnecessary costs and time in attending a health structure. CMVs could receive additional training regarding respiratory infections to distinguish between serious signs of pneumonia (e.g. increased respiratory rate) and those of common cold viruses. This strategy has support in the literature where CHWs have been able to diagnose and treat pneumonia in Nepal [22] and in Uganda [23]. To offer appropriate treatment to RDT-negative patients with fever, adapting the treatment criteria and training CMVs in diagnosing and treating for example pneumonia and diarrhoea, should also be considered. In Zambia, a cluster-randomized controlled trial among CHWs showed great potential for CHWs to manage both malaria and pneumonia at community level. Over 68% of the children treated for simple pneumonia by CHWs received early and appropriate treatment, compared to 13.3% of the children who were referred to the health centre [24]. This strategy would, however, put heavier responsibilities on CMVs that would require additional training, supervision and support.

There are some encouraging results from this study. Previous research shows that CHWs are capable of using and interpreting RDTs [25,26]. The high positivity rate in this study suggests that the CMVs are following the correct indications for testing, treating patients with simple malaria. The study also shows that the CMVs are, at least, suggesting referral for patients with signs of severe malaria and with negative RDTs. There is a possibility that the high RDT-positivity rate is due to over-diagnosing of malaria, and that patients might not have received a referral advice, though noted as referred. With increased supervision this could be prevented.

This retrospective study had several limitations. The short study duration may have been influenced by disease patterns, disease variations and seasonal activities such as farming, affecting caretakers' behaviour. The low number of referral completions did not allow for a proper analysis of outcome and timeliness of referrals, as well as for impact of distance from villages to referral structures. As analysis of data was done through record reviews it could not be verified that all referred RDT-positive patients had signs of severe malaria. Analysis of signs of severe disease among RDT-negative patients could also not be carried out. The record review was done in two steps: comparison of the two records (CMV recording forms and CMV referral follow up register) and encoding of this data was done in the field. The data analysis was done at Karolinska Institutet, and there is a possibility of misinterpretation of data. With only two CMV supervisors responsible for data collection and direct CMV supervision, it is possible that referred patients were not properly entered in the CMV recording forms. There is also a risk that patients were missed due to variations in their own or their caretaker's name. Using three different data-clerks in the field for encoding might have increased data errors. Finally, a qualitative, in-depth study, interviewing CMVs, health personnel and patients could give a better picture of the underlying reasons for non-compliance of referrals.

## Conclusions

This study showed that most RDT-negative patients with fever seen by CMVs in a community-based programme in Sierra Leone did not arrive at a referral structure. This finding has prompted a review of the programme by MSF. To map the underlying reasons for non-completion of referrals and to define a suitable strategy for improving the referral completion in MSF's community-based programme, additional research is needed. Similar community-based malaria programmes should assess their referral-completion rates to ensure that patients with severe malaria or other illnesses receive treatment.

## Competing interests

The authors declare that they have no competing interests.

## Authors' contributions

AT designed the study, analysed the data and wrote the paper with major contributions of the other authors. AM approved the study and initiated discussions around study objectives. TR contributed with the study design and gave input to the study protocol and manuscript. MK entered and analysed the data and supervised the fieldwork. JvS, SP and MdS monitored the study progress and contributed significantly to improve the manuscript. All authors read and approved the final draft.

## References

[B1] WHO World malaria report 2010 2010 Geneva: World Health Organization

[B2] WHOSIS detailed database search Under-five mortality rate (probability of dying by age 5 per 1000 live births), both sexes, Sierra Leone http://apps.who.int/whosis/data/Search.jsp accessed 18 December 2010

[B3] WHO Sierra Leone Health Profile 2010 http://www.who.int/gho/countries/sle.pdf accessed 19 December

[B4] Checci F Roddy P Kamara S Williams A Morineau G Wurie AR Hora B de Lamotte N Baerwaldt T Heinzelmann A Danks A Pinoges L Oloo A Durand R Ranford-Cartwright L de Smet M Evidence basis for antimalarial policy change in Sierra Leone: five *in vivo *efficacy studies of chloroquine, sulphadoxine-pyrimethamine and amodiaquine Trop Med Int Health 2005 10 146 153 10.1111/j.1365-3156.2004.01367.x 15679557

[B5] WHO Guidelines for the treatment of malaria (second edition) 2010 Geneva: World Health Organization

[B6] MSF Full prescription. Better malaria treatment for more people, MSF's experience 2008 Brussels: Médecins Sans Frontières

[B7] MSF Sierra Leone Annual Medical Report 2008 (internal document) 2009 Brussels: Médecins Sans Frontières Operational Centre Brussels

[B8] Simba DO Warsame M Kimbute O Kakoko D Petzold M Tomson G Premji Z Gomes M Factors influencing adherence to referral advice following pre-referral treatment with artesunate suppositories in children in rural Tanzania Trop Med Int Health 2009 14 775 783 10.1111/j.1365-3156.2009.02299.x 19497077

[B9] Winch PJ Bagayoko A Diawara A Kane M Thiero F Gilroy K Daou Z Berthe Z Swedberg E Increases in correct administration of chloroquine in the home and referral of sick children to health facilities through a community-based intervention in Bougouni District, Mali Trans R Soc Trop Med Hyg 2003 97 481 490 10.1016/S0035-9203(03)80001-9 15307407

[B10] Kallander K Tomson G Nsungwa-Sabiiti J Senyonjo Y Pariyo G Peterson S Community referral in home management of malaria in western Uganda: a case series study BMC Int Health Hum Rights 2006 6 2 10.1186/1472-698X-6-2 16539744 PMC1434779

[B11] Sievers AC Lewey J Musafiri P Franke MF Bucyibaruta BJ Stulac SN Rich ML Karema C Daily JP Reduced paediatric hospitalizations for malaria and febrile illness patterns following implementation of a community-based malaria control programme in rural Rwanda World Hosp Health Serv 2008 44 28 35 19370834

[B12] Elmardi KA Malik EM Abdelgadir T Ali SH Elsyed AH Mudather MA Elhassan AH Adam I Feasibility and acceptability of home-based management of malaria strategy adapted to Sudan's conditions using artemisinin-based combination therapy and rapid diagnostic test Malar J 2009 8 39 10.1186/1475-2875-8-39 19272157 PMC2660358

[B13] Tiono AB Kaboré Y Traoré A Convelbo N Pagnoni F Sirimba SB Implementation of Home based management of malaria in children reduces the work load for peripheral health facilities in a rural district of Burkina Faso Malar J 2008 7 201 10.1186/1475-2875-7-201 18834504 PMC2570683

[B14] Kidane G Morrow RH Teaching mothers to provide home treatment of malaria in Tigray, Ethiopia: a randomised trial Lancet 2000 356 550 555 10.1016/S0140-6736(00)02580-0 10950232

[B15] Lemma H Byass P Desta A Bosman A Costanzo G Toma L Fottrell E Marrast AC Ambachew Y Getachew A Mulure N Morrone A Bianhi A Barnabas GA Deploying artemether-lumefantrine with rapid testing in Ethiopian communities: impact on malaria morbidity, mortality and healthcare resources Trop Med Int Health 2010 15 241 250 10.1111/j.1365-3156.2009.02447.x 19961564

[B16] Hopkins H Talisuna A Whitty CJ Staedke SG Impact of home-based management of malaria on health outcomes in Africa: a systematic review of the evidence Malar J 2007 6 134 10.1186/1475-2875-6-134 17922916 PMC2170444

[B17] Peterson S Nsungwa-Sabiiti J Were W Nsabagasani X Magumba G Nambooze J Mukasa G Coping with paediatric referral—Ugandan parents' experience Lancet 2004 363 1955 1956 10.1016/S0140-6736(04)16411-8 15194257

[B18] Kalter HD Salgado R Moulton LH Nieto P Contreras A Egas ML Black RE Factors constraining adherence to referral advice for severely ill children managed by the Integrated Management of Childhood Illness approach in Imbabura Province, Ecuador Acta Paediatr 2003 92 103 110 10.1111/j.1651-2227.2003.tb00478.x 12650309

[B19] Okeke TA Improving malaria recognition, treatment and referral practices by training caretakers in rural Nigeria J Biosoc Sci 2010 42 325 339 10.1017/S0021932009990484 19941680

[B20] Okeke TA Uzochukwu BS Improving childhood malaria treatment and referral practices by training patent medicine vendors in rural south-east Nigeria Malar J 2009 8 260 10.1186/1475-2875-8-260 19930561 PMC2784476

[B21] Haines A Sanders D Lehmann U Rowe AK Lawn JE Jan S Walker DG Bhutta Z Achieving child survival goals: potential contribution of community health workers Lancet 2007 369 2121 2131 10.1016/S0140-6736(07)60325-0 17586307

[B22] Dawson P Pradhan Y Houston R Karki S Poudel D Hodgins S From research to national expansion: 20 years' experience of community-based management of childhood pneumonia in Nepal Bull World Health Organ 2008 86 339 343 10.2471/BLT.07.047688 18545735 PMC2647452

[B23] Kallander K Tomson G Nsabagasani X Sabiiti JN Pariyo G Peterson S Can community health workers and caretakers recognise pneumonia in children? Experiences from western Uganda Trans R Soc Trop Med Hyg 2006 100 10 956 963 10.1016/j.trstmh.2005.11.004 16455119

[B24] Yeboah-Antwi K Pilingana P Macleod WB Semrau K Siazeele K Kalesha P Hamainza B Seidenberg P Mazimba A Sabin L Kamholz K Thea DM Hamer DH Community case management of fever due to malaria and pneumonia in children under five in Zambia: a cluster randomized controlled trial PLoS Med 2010 7 e1000340 10.1371/journal.pmed.1000340 20877714 PMC2943441

[B25] Hawkes M Katsuva JP Masumbuko CK Use and limitations of malaria rapid diagnostic testing by community health workers in war-torn Democratic Republic of Congo Malar J 2009 8 308 10.1186/1475-2875-8-308 20028563 PMC2804690

[B26] Yasuoka J Poudel KC Poudel-Tandukar K Nguon C Ly P Socheat D Jimba M Assessing the quality of service of village malaria workers to strengthen community-based malaria control in Cambodia Malar J 2010 9 109 10.1186/1475-2875-9-109 20412600 PMC2873522

